# Wine Consumer Studies: Current Status and Future Agendas

**DOI:** 10.12688/f1000research.146631.1

**Published:** 2024-03-27

**Authors:** Vageesh Neelavar Kelkar, Jyothi Mallya, Valsaraj Payini, Vasanth Kamath

**Affiliations:** 1 Food and Beverage Services, Welcomgroup Graduate School of Hotel Administration, Manipal Academy of Higher Education, Manipal, Karnataka, 576104, India; 2 Library, Welcomgroup Graduate School of Hotel Administration, Manipal Academy of Higher Education, Manipal, Karnataka, 576104, India; 3 Food and Beverage Services, Welcomgroup Graduate School of Hotel Administration, Manipal Academy of Higher Education, Manipal, Karnataka, 576104, India; 4 Operations Management, T A Pai Management Institute , Manipal Academy of Higher Education, Manipal, India, 576104, Manipal, Karnataka, 576104, India

**Keywords:** bibliometrics, systematic review, integrative review, environmental, economic, sociocultural, sustainability

## Abstract

**Background:**

As wine has become more than just a drink, exploring wine consumer studies provides a better understanding of various factors that shape the wine industry. Therefore, this paper aims to review and map the landscape of wine consumer literature using bibliometric analysis and systematic review. It identifies the key areas, clusters, antecedents, mediators, moderators, and outcomes to propose the framework for future research directions.

**Methods:**

This study adopts an integrative review approach: a bibliometric and systematic review. The data for this study were retrieved from the Scopus database. While the bibliometric analyses are conducted using VoSviewer software, a systematic review is conducted using a content analysis approach.

**Results:**

Four main topics in the extant wine consumer literature are identified: sustainability and wine, wine preferences and choice, wine consumer behavior, and wine consumer insights. The five critical areas of wine consumers’ literature recognized are decision-making, consumer preferences, consumer behavior, segmentation, and consumer involvement. This study also recognizes theoretical and methodological advancements in the wine consumer literature.

**Conclusions:**

The findings contribute to advancing knowledge development, identifying research gaps and shedding light on future research in the wine consumer domain. The results offer practical insight for wine industry stakeholders, researchers, and influencers.

## 1. Introduction

Since ancient times, wine has been admired as a cultural icon, a symbol of sophistication, and a source of enjoyment. Beyond its gastronomic appeal, the world of wine is a fascinating domain where science, culture, and consumer behaviour intertwine ( Mitchell *et al.*, 2009; Mouret *et al.*, 2013). Consequently, over the past two decades, wine consumers’ preferences and decision-making processes have attracted much interest from researchers and industry professionals alike ( Aqueveque, 2023; Moscovici *et al.*, 2022; Payini, 2021; Payini *et al.*, 2022; Vecchio *et al.*, 2023). Accordingly, much research has been done on how consumer choose wines ( Wright *et al.*, 2023). Therefore, it is essential to understand the factors that influence wine preferences, consumption patterns, and selections. Though traditional research methodologies, such as bibliometric analysis ( Martinho, 2021; Weatherbee *et al.*, 2019) and systematic reviews ( Campo *et al.*, 2022; Carollo *et al.*, 2022; Schäufele and Hamm, 2017), have long been employed to analyze and synthesize knowledge within wine business domains, an integrating these approaches offers a unique opportunity to gain comprehensive insights into wine consumers’ behavior. It is found that most of the reviews pertain to particular theme, such as wine tourism experience ( Gómez *et al.*, 2019; Kotur, 2023), wine consumption ( Wright *et al.*, 2023), sustainability in wine industry ( Nave *et al.*, 2021), sustainable wine ( Maesano *et al.*, 2019), and willingness to pay ( Schäufele and Hamm, 2017). Additionally, none of these reviews attempts to encompass the entirety of wine consumption. Further, there are no studies that examined the conceptual and intellectual configuration latent in this emergent research field. Such omissions motivated the researchers to combine quantitative and qualitative methods in order to consolidate the existing literature and provide a road map for future research. Therefore, this integrative review aims to provide the current state of knowledge of wine consumers’ behavior. The findings of this study are helpful for researchers, practitioners, and policymakers.

Bibliometrics, as a quantitative analysis of scientific publications, provides a valuable means to map and assess the existing body of research in a particular field ( Mukherjee *et al.*, 2022a). By systematically identifying and analyzing relevant publications, bibliometrics enables researchers to uncover key trends, knowledge gaps, and emerging research areas. In the context of wine consumers’ behavior, bibliometric analyses can shed light on the evolution of wine consumer research, influential authors, and predominant themes or theories. It serves as a foundational step in understanding the overall landscape of research on wine consumers ( Donthu *et al.*, 2021).

On the other hand, systematic reviews offer a rigorous and structured approach to identifying patterns, discrepancies, and evidence-based findings ( Mulrow, 1994). By employing predefined inclusion and exclusion criteria, systematic reviews ensure objectivity and reproducibility in selecting relevant studies ( Moher *et al.*, 2009). In the context of wine consumers’ behavior, systematic reviews can help identify the factors influencing consumer choices, preferences, and decision-making processes. By combining the quantitative analysis of bibliometrics with the qualitative synthesis of systematic reviews, this study offers a holistic view of the existing body of wine consumer literature. It allows the identification of theoretical and methodological research gaps.

This integrative review addresses several critical questions, such as the following: What are the publication trends of wine consumer literature? What is the performance of research constituents, such as authors, journals, and articles? What are the critical areas and clusters of wine consumer literature? What are the antecedents, mediators, and outcomes of wine consumption? Which theoretical frameworks have been most frequently employed to understand wine consumers? How can future research directions be shaped to enhance our understanding of wine consumers’ behavior? By combining bibliometric analysis and systematic review methodology, this study seeks to contribute to the wine consumer knowledge base by identifying research gaps and proposing potential areas for future investigation. The proposed framework helps to identify the underlying gaps in a research domain across theory development and methods.

## 2. Methods

To achieve the objectives set in this research, a bibliometric analysis was conducted using VOSViewer. In addition, a systematic literature review was conducted as the bibliometric analysis alone is considered insufficient to explore and further the research practices in the field ( Ali *et al.*, 2022).

### 2.1 Data source

The Scopus database was the data source for this study. Scopus indexes content from over 25,000 active titles and 7,000 publishers—all rigorously vetted and selected by an independent review board. It is the largest database of peer-reviewed literature and is widely used for similar studies ( Donthu *et al.*, 2021).

### 2.2 Search criteria

The keywords “wine” and “consum*” were searched in the article title head of the Scopus database. After restricting to the “Business, Management, and Accounting” subject area, 317 articles were obtained. After excluding editorials, book chapters, and non-English articles, the final dataset extracted was 262 articles (Mallya, Jyothi, *et al*., 2024). The final query string is as follows: ((TITLE (consum*) AND TITLE (wine)) AND (LIMIT-TO (SUBJAREA, “BUSI”)) AND (LIMIT-TO (DOCTYPE, “ar”)) AND (LIMIT-TO (LANGUAGE, “English”)) AND (LIMIT-TO (SRCTYPE, “j”)).

### 2.3 Software used for the analysis

VoSviewer, a free software available is used to conduct various analyses. It is a popular and frequently used software by researchers for constructing and visualizing bibliometric networks. These networks may include journals, contributors, or individual publications, and they can be built based on citation, bibliographic coupling, cocitation, or coauthorship relations. This software also offers text mining functionality that can be used to construct and visualize co-occurrence networks of important terms extracted from a body of scientific literature ( van Eck and Waltman, 2010).

### 2.4 Types of analyses

Bibliometrics analyses, a branch of library science, have been used in various disciplines, including consumer behavior research ( Baber *et al*., 2023; Haba *et al*., 2023). Utilizing quantitative methods, it analyses bibliographic information to derive insightful conclusions ( Mukherjee *et al*., 2022b). This approach is more advantageous ( Mukherjee *et al*., 2022a), making it more appropriate for the current study. It supports a huge corpus of data and involves a variety of bibliographic metrics. Its quantitative character of analyses yields impartial results. Additionally, the networks and graphs produced by this program allow users higher visibility of the data points ( Donthu *et al*., 2021).

However, bibliometric approaches have certain limitations, such as making qualitative claims about research based on quantitative data ( Wallin, 2005). Quantitative metrics such as citation counts, h-indexes, and journal impact factors are typically utilized in bibliometric analyses. Although these metrics provide a quantitative evaluation of research output, they may not capture the complexity and quality of scientific work. Advancing theory and methodology frequently requires a deeper understanding of research content, context, and impact, which bibliometric indicators may not adequately reflect. This dependency may hinder the study’s findings. Therefore, to fill this gap, the current study also systematically reviews selected articles to support a few qualitative assumptions.

**2.4.1 Bibliometric analyses**

2.4.1.1 Descriptive analysis

The descriptive analysis includes the number of publications in a given dataset over a specific period. It provides an overview of the volume of research output in a particular field or topic. The annual and last four decades of publication growth were analyzed.

2.4.1.2 Performance analyses

In bibliometric studies, performance analysis refers to assessing the research output of people (for example, authors), organizations (institutions and funding agencies), nations, sources, and documents using bibliometric data. It includes information about the quantity and quality of research constituents, such as the number of publications, citations, impact factor of journals, and H-index. Thus, the pattern in the research constituents (journals, articles, and authors) was also explored as recommended ( Donthu *et al.*, 2021). The performance of the various research constituents, such as journals, authors, articles, and countries, was assessed regarding productivity, influence, and authorship structure. The multiple matrices used are total publications (TP), total citations (TC), number of cited publications (NCP), total citations per publication (TC/TP), number of active years (NAY), productivity per active year (PAY), number of contributing authors (NCA) and h and g indexes ( Ali *et al*., 2022). These analyses were conducted using MS Excel software.

2.4.1.3 Science mapping

Science mapping is a bibliometric technique that uses patterns of citation, cocitation, and coauthorship in the scientific literature to visually demonstrate subjects’ structure and evolution. It provides a thorough overview of the research landscape within a specific field by enabling researchers to spot clusters of linked research, notable authors, and important research subjects. This analysis includes cocitation mapping, bibliographic coupling analysis, and keyword co-occurrence. The current study uses keyword-cooccurrence and bibliographic coupling analysis to uncover the domains and themes of the wine consumption literature landscape. While keyword co-occurrence analysis examines the frequency of keywords or terms within a set of documents and identifies co-occurrence patterns, bibliographic coupling analysis identifies relationships between documents based on shared references. Both of these methods help identify clusters of related documents, identify central or influential documents, and analyze the relationships between studies in a given field.

**2.4.2 Systematic review**

Despite the clear advantages of bibliometric analyses, it is important to remember that bibliometric techniques alone are insufficient for advancing theory and practice ( Ali *et al.*, 2022). Each review method contributes to the overall body of knowledge and comprehension, provided that they form knowledge theoretically ( Donthu *et al.*, 2021; Fan *et al.*, 2022). Content analysis has been successfully used in wine research as a research method ( Bonn *et al.*, 2018; Carollo *et al.*, 2022; Nave *et al.*, 2021; Weatherbee *et al.*, 2019). The primary purpose of the content analysis was to synthesize the extant wine consumer literature to gain a comprehensive understanding of it. Furthermore, the content analysis aims to understand the wine consumption literature’s theoretical and methodological advancement and propose research frameworks for future studies.

## 3. Results and discussion

### 3.1 Descriptive statistics

**Figure 1.**
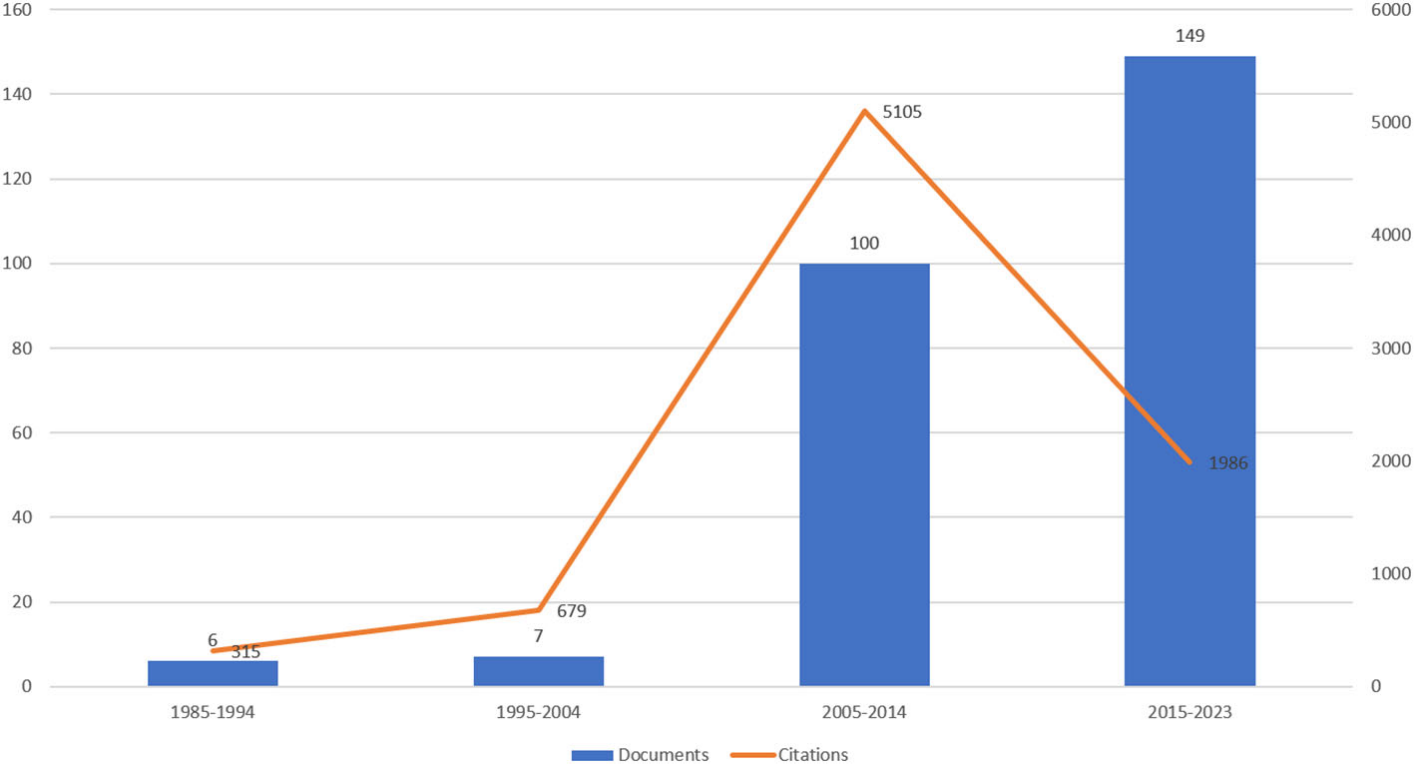
Number of documents and citations for four different periods. Source: Authors own.

**Figure 2.**
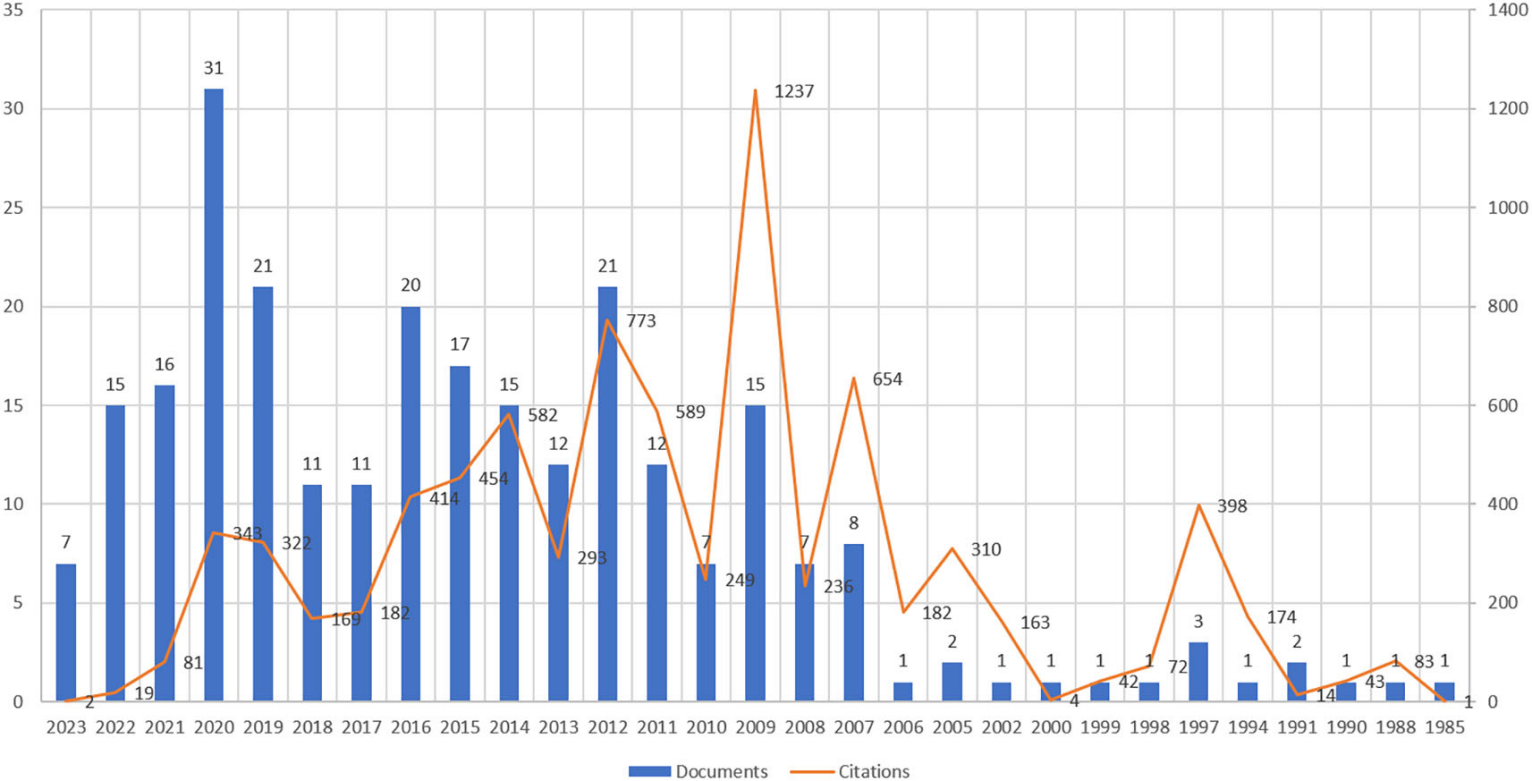
Yearwise number of documents and citations. Source: Authors own.

The first research question was to understand the research publication trends. To this effect, descriptive analysis was conducted ( Figures 1 and 2). While Figure 1 presents the direction of wine consumption literature for the last four decades, Figure 2 reveals the annual growth of publications and citations. According to Moed *et al.* (2004), the count of publications’ productivity and the citation counts represent academic influence and impacts ( Meyer *et al.*, 2018).

The data presented in Figure 1 highlight a significant increase in research activity over approximately four decades within this subject area. Specifically, the number of documents produced has notably increased, from only six in the earliest period (1985-1994) to 149 in the most recent period (2015-2023). These data suggest a growing interest and investment in this field, potentially due to the wine consumer study’s increasing importance and relevance for the hospitality industry. However, there have been fluctuations between different periods regarding citation trends. For example, the average number of citations in the third period was significantly more (51 citations per document) than in the fourth period (13 citations per document). It is also worth noting that the most recent period (2015-2023) shows a high volume of document production (149) but a relatively low citation count (1986), which may be attributed to the recency of the research and its ongoing evaluation by the wine consumers’ research community.

Figure 2, the year wise distribution of the publications, shows somewhat inconsistent growth regarding the number of articles. For example, 2020 witnessed the highest number of publications (31 articles), followed by 2019 and 2012, with 21 articles each. However, the articles published in 2009 received the highest number of citations, nearly 15% of the total citations across 15 documents. Thus, it can be inferred that 2020 and 2009 are the most productive and impactful years in wine consumers’ literature.

### 3.2 Performance analysis

The second research objective was to analyze the performance of various research constituents, such as authors, journals, articles, and countries. For this, citation analysis was used.

3.2.1 Most productive authors

The top ten most productive authors were identified based on the number of publications (at least five articles) and citations (a minimum of 75 citations). Table 1 demonstrates that Johan Bruwer, with 27 papers and 1417 citations, is the most prolific author, followed by Nelson Barber and Tim H. Dodd, who have eight articles. Tim H. Dodd and Nelson Barber received 492 and 314 citations, respectively. Bruwer Johan has been active in the wine consumer subject domain for 12 years (NAY=12) and has the highest h-index of 19. At the same time, Anthony Saliba J. emerged as one of the most prolific authors, receiving 320 citations across five documents. Steve Goodman has the highest TC/CP ratio, indicating that the four cited publications contributed by the author carry an average of 73.50 citations.

**Table 1.** Top ten productive authors.

	TP	SOA	COA	CP	TC	TC/TP	TC/CP	NAY	PAY	H
Bruwer J	27	0	27	26	1417	52.48	54.50	12	118.08	19
Barber N.	8	0	8	8	314	39.25	39.25	4	78.50	7
Dodd T H	8	0	8	8	492	61.50	61.50	6	82.00	7
Agnoli	6	0	6	6	113	18.83	18.83	5	22.60	4
Kelly	6	0	6	6	86	14.33	14.33	5	17.20	5
Agnoli L	5	0	5	5	113	22.60	22.60	5	22.60	4
Begalli	5	0	5	5	108	21.60	21.60	5	21.60	4
Capatillo	5	0	5	5	108	21.60	21.60	5	21.60	4
Corsi A M	5	0	5	4	175	35.00	43.75	4	43.75	4
Goodman	5	1	4	4	294	58.80	73.50	3	98.00	5
Saliba J	5	0	5	5	320	64.00	64.00	3	106.67	5

Notes: TP = total publications; SoA = sole authored publications; CoA = coauthored publications; CP = cited publication; TC = total citations; TC/TP = average citations per publication; TC/CP = average citations per cited publication; NAY = number of active years; PAY = productivity per active year; H = h-index.

Source: Author’s own.

3.2.2 Most influential articles

An analysis of the top 15 most cited articles in wine consumer studies based on the number of citations was conducted. Table 2 provides the total number of citations and average citations per year of the top 15 articles in the domain of wine consumption literature. As evident, the article “The Hedonic Nature Of Wine Tourism Consumption: An Experiential View” ( Bruwer and Alant, 2009) was an influential publication with 252 citations. Bruwer and Alant used the experiential view of consumption to better understand wine tourists’ motivation. The other most cited works include articles titled “Consumer Attitudes Regarding Environmentally Sustainable Wine: An Exploratory Study of The New Zealand Marketplace” ( Forbes *et al.*, 2009). and “Differential Effects of Experience, Subjective Knowledge, And Objective Knowledge on Sources of Information Used in Consumer Wine Purchasing” ( Dodd *et al.*, 2005). However, the articles titled “Millennial Generation Attitudes toward Sustainable Wine: An Exploratory Study on Italian Consumers” ( Pomarici and Vecchio, 2014) and “Exploring Environmental Consciousness and Consumer Preferences for Organic Wines Without Sulfites” ( D’Amico *et al.*, 2016) are the fastest growing articles in terms of influence, with an average of 21 and 19 citations per year, respectively.

**Table 2.** Most cited articles.

	Authors	Title	Year	Source Title	Citations
1	( Cortez *et al.*, 2009)	Modeling wine preferences by data mining from physicochemical properties	2009	Decision Support Systems	784
2	( Lynch Jr and Ariely, 2000)	Wine online: Search costs affect competition on price, quality, and distribution	2000	Marketing Science	641
3	( Getz and Brown, 2006)	Critical success factors for wine tourism regions: A demand analysis	2006	Tourism Management	498
4	( Charters and Ali-Knight, 2002)	Who is the wine tourist?	2002	Tourism Management	426
5	( Orth and Malkewitz, 2008)	Holistic package design and consumer brand impressions	2008	Journal of Marketing	419
6	( Beverland, 2006)	The 'real thing': Branding authenticity in the luxury wine trade	2006	Journal of Business Research	388
7	( Ariely and Levav, 2000)	Sequential choice in group settings: Taking the road less traveled and less enjoyed	2000	Journal of Consumer Research	337
8	( Mason and Paggiaro, 2012)	Investigating the role of festivalscape in culinary tourism: The case of food and wine events	2012	Tourism Management	262
9	( Bruwer and Alant, 2009a)	The hedonic nature of wine tourism consumption: An experiential view	2009	International Journal of Wine Business Research	259
10	( Landon and Smith, 1997)	The use of quality and reputation indicators by consumers: The case of Bordeaux wine	1997	Journal of Consumer Policy	244
11	( Gupta *et al.*, 2004, p. 200)	An empirical study of consumer switching from traditional to electronic channels: A purchase-decision process perspective	2004	International Journal of Electronic Commerce	234
12	( Yuan and Jang, 2008)	The effects of quality and satisfaction on awareness and behavioral intentions: Exploring the role of a wine festival	2008	Journal of Travel Research	230
13	( Forbes *et al.*, 2009)	Consumer attitudes regarding environmentally sustainable wine: an exploratory study of the New Zealand marketplace	2009	Journal of Cleaner Production	226
14	( Labroo *et al.*, 2008)	Of frog wines and frowning watches: Semantic priming, perceptual fluency, and brand evaluation	2008	Journal of Consumer Research	214
15	( Quester and Smart, 1998)	The influence of consumption situation and product involvement over consumers' use of product attribute	1998	Journal of Consumer Marketing	208
16	( Dodd *et al.*, 2005)	Differential Effects of Experience, Subjective Knowledge, and Objective Knowledge on Sources of Information used in Consumer Wine Purchasing	2005	Journal of Hospitality and Tourism Research	205
17	( Johnson and Bruwer, 2007)	Regional brand image and perceived wine quality: The consumer perspective	2007	International Journal of Wine Business Research	200

Notes: TC = total citations; ACY = Average citations per year.

Source: Author’s own.

3.2.3 Journal impact

The most influential journal was identified based on a number of publications and citations. The journals published at least five articles, and 100 citations were included in the analysis. The source impact (productivity and influence) analysis helps understand the distribution of core journals in the research area. Table 4 shows the top seven journals in the research area sorted by the number of publications and citations. The h-index 26 for the International Journal of Wine Business Research (IJWBR) indicates that IJWBR has at least 26 documents, each receiving at least 26 citations. IJWBR is the only journal that comprehensively covers business disciplines, continents, and countries on all topics connected to wine consumer literature, such as perspectives on alcoholic beverages such as beer, craft beer, and spirits. Thus, it can be concluded that wine consumption is widely acknowledged in wine business research literature. Following IJWBR, the British Food Journal (BFJ), the Journal of Food Products Marketing (JFPM), and the Journal of Cleaner Production (JCP) are positioned as the second and the third most impactful journals in this domain ( Tables 3 and 4). These results also indicate that wine consumption is well documented in food science, food product marketing, and environmental/sustainability research literature.

**Table 3.** Source productivity.

Source title	TP	NPC	NAY	PAY
IJWBR	73	66	17	4
BFJ	53	51	18	3
JFPM	16	16	9	2
JCP	13	13	8	2
APJML	6	6	6	1
IJHM	6	6	5	1
CHQ	5	4	3	2

Notes: TP = total publications; NPC = Number of Cited publications; NAY = number of active years; PAY = productivity per active year.

Source: Author’s own.

**Table 4.** Source influence.

Source title	TC	TC/TP	TC/NPC	TC/NAY	H-Index
IJWBR	2264	31	34	133	26
BFJ	1482	28	29	82	20
JFPM	325	20	20	36	10
JCP	1157	89	89	145	10
APJML	246	41	41	41	6
IJHM	119	20	20	24	5
CHQ	136	27	34	45	2

Notes: TC = total citations; TC/TP = average citations per publication; NPC = Number of Cited publications; TC/NPC = Average citations per cited publications; NAY = number of active years; PAY = productivity per active year; TC/NAY=Citations per active years, H-index.

Source: Author’s own.

3.2.4 Top contributing countries

The following is a list of the top ten countries according to the nationality of the corresponding author. Table 5 presents the total publications, total cited publications, total citations (TC), average citations per publication, average citations per cited publication, number of active years, productivity per active year, and H-index for the ten leading countries in the wine consumption literature. According to this table, the most influential country is the United States of America, with 21 active years, 67 publications, and 2376 citations. However, in terms of the H-index, Australia topped the list with an H-index of 27. Additionally, the highest number of publications are also from the United States of America. Interestingly, despite having eight active years and an H-index, Canada topped the list regarding average citations per publication and cited publications, followed by New Zealand.

**Table 5.** Top 10 countries.

No.	Country	TP	TCP	TC	TC/TP	TC/TCP	NAY	PAY	H
1	United States	67	64	2376	35	37	21	3	26
2	Australia	57	56	2335	41	42	17	3	27
3	Italy	47	43	1186	25	28	15	3	19
4	France	26	24	570	22	24	12	2	12
5	Spain	19	18	390	21	22	12	2	10
6	United Kingdom	15	15	650	43	43	12	1	12
7	South Africa	13	11	185	14	17	8	2	6
8	Canada	10	10	558	56	56	8	1	7
9	Germany	9	8	244	27	31	6	2	7
10	New Zealand	9	9	450	50	50	7	1	9

Notes: TP = total publications; TCP = total cited publications; TC = total citations; TC/TP = average citations per publication; TC/CP = average citations per cited publication; NAY = number of active years; PAY = productivity per active year; H = h index.

Source: Author’s own.

### 3.3 Science mapping

The third objective of this study was to identify the critical areas and distinct clusters of wine consumption literature. The study uses keyword co-occurrence analysis and bibliometric coupling analysis to achieve this.

3.3.1 Keyword co-occurrence analysis

**Figure 3.**
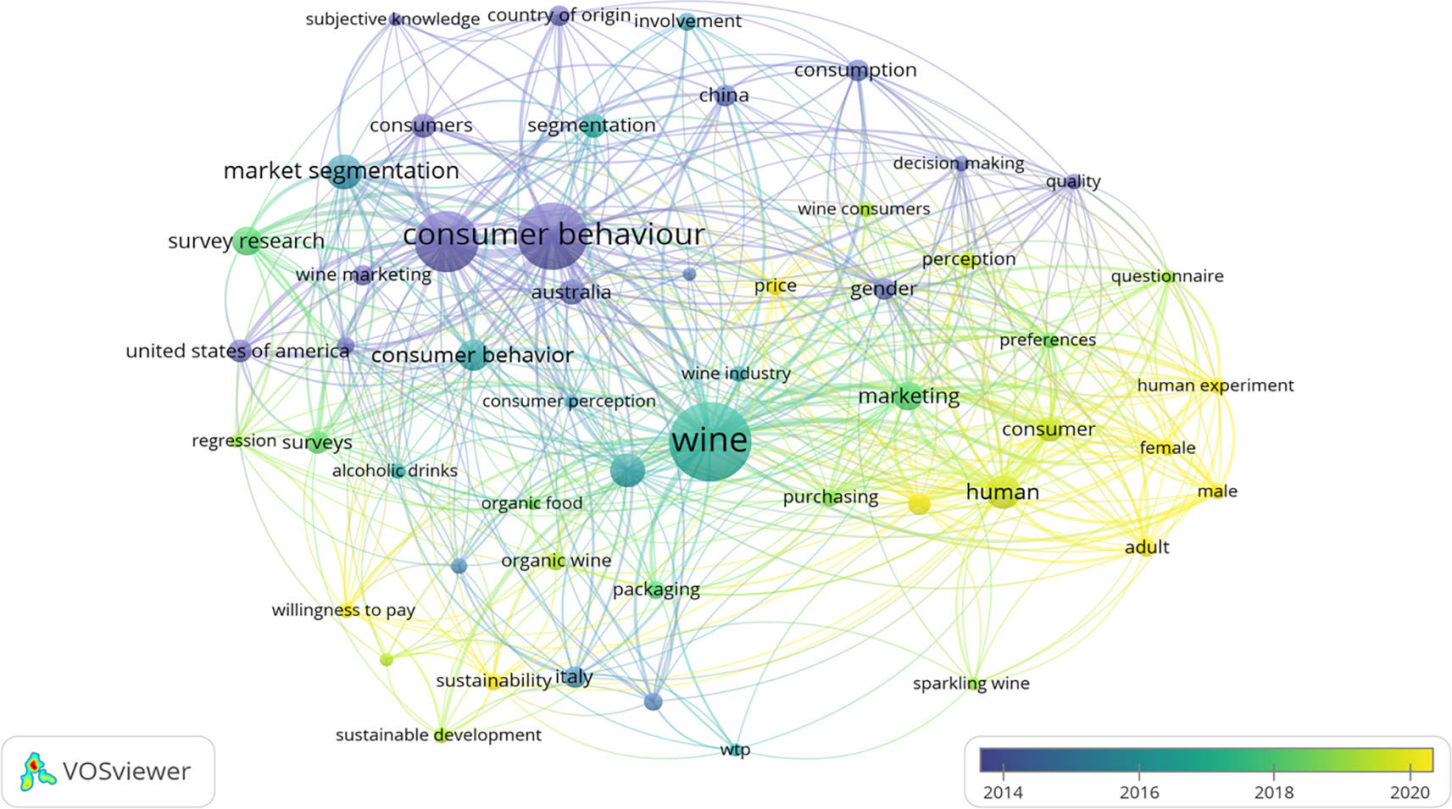
Overlay visualization of the most frequent keywords where the score of the item is the time since publication. Source: Generated by the authors using VOS-viewer software.

Keyword co-occurrence analysis refers to examining the co-occurrence of keywords in scientific publications and constructing a network of keywords based on their relationships. Identifying the frequency of keywords could uncover potential future research opportunities. Therefore, the current study conducts keyword co-occurrence analysis using VoSviewer software. The minimum number of occurrences was set to five. VOSviewer provides various visualization techniques to represent coword networks, such as network and overlay visualization ( Figure 3). The keyword co-occurrence analysis resulted in six clusters. The keyword co-occurrence analysis resulted in five critical areas of wine consumer literature: decision making, consumer preferences, consumer behavior, segmentation, and consumer involvement. The clusters and items are presented in Table 6.

**Table 6.** Clusters based on keyword co-occurrence analysis.

Decision-making (15 items)	Consumer preferences (13 items)	Consumer behavior (12 items)	Segmentation (10 items)	Consumer involvement (3 items)
human	wine	consumer behavior	consumer behavior	China
marketing	wine consumption	wines	segmentation	consumption
consumer	Italy	market segmentation	organic wine	involvement
consumer attitude	wine marketing	survey research	Perception	
gender	consumer preferences	Australia	price	
adult	packaging	consumers	wine consumers	
purchasing	sustainability	surveys	wine industry	
decision making	alcoholic drinks	united states of America	consumer perception	
female	environment	country of origin	consumption behavior	
human experiment	sustainable development	marketing strategy	organic food	
male	willingness to pay	regression		
preferences	consumer choice	subjective knowledge		
quality	wtp			
questionnaire				
sparkling wine				

Source: Author’s own.

The size of the nodes in Figure 3 reflects the occurrence of using the terms. Overlay visualization, a feature in VOSviewer that allows the classification of keywords based on a time scale, is conducted. It is evident that the word “wine/wines” is the most used keyword (145 occurrences), followed by consumer behavior/behavior (96 occurrences). The other keywords are market segmentation (23 occurrences), human (21 occurrences), wine consumption (21 occurrences), and marketing (16 occurrences). Keywords that appeared more than 10 times are consumers, segmentation, United States of America, China, consumer attitude, consumption, gender, and Italy. The items are colored differently according to publishing year (average for the cluster). This study highlights phrases that debuted recently (the average publication year was 2020) in a brighter yellow. A color bar displayed at the corner has the same explanation; the scores of the items are decided by the publishing date ( Figure 3). According to Figure 3, the most recent topics studied were sustainable development, sustainability, willingness to pay, organic wine, and price.

3.3.2 Bibliographic coupling analysis

**Figure 4.**
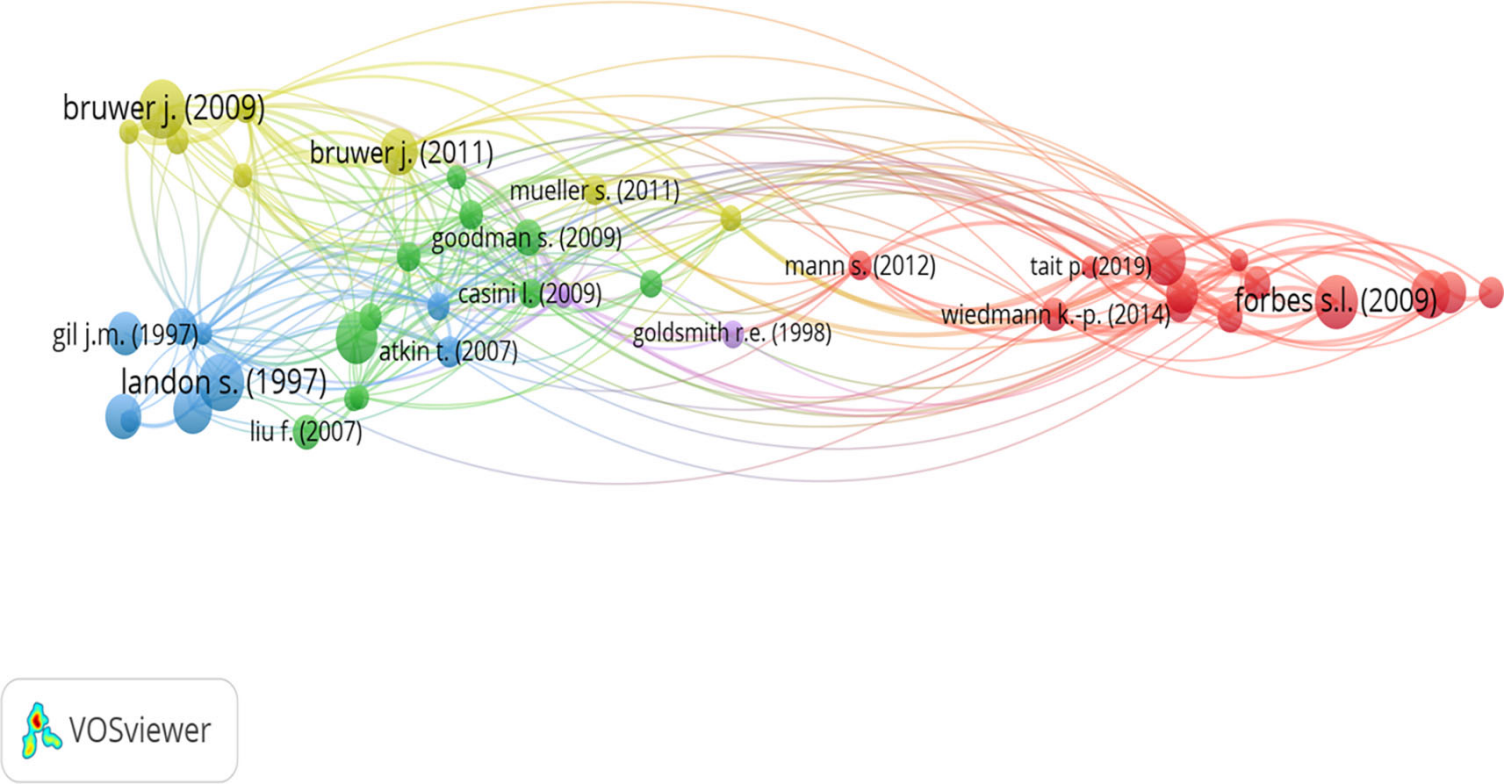
Clusters of wine consumer literature. Source: Generated by the authors using VOS-viewer software.

Bibliographic coupling is a measure of similarity that employs citation analysis to build a relationship of similarity between documents. It occurs when two works’ bibliographies reference the same third work. This indicates that there is a possibility that the two works address a similar topic ( Weinberg, 1974). It is helpful in various fields since it helps researchers find related past research. Bibliographic coupling uniquely contributes to the measurement of centeredness, with increasing coupling between the precenter and postcenter periods ( Youtie *et al.*, 2013). Bibliographic coupling analysis is also helpful in identifying clusters of related articles. Therefore, this study embraces bibliographic coupling analysis to identify the different clusters of wine consumption literature based on a minimum of 50 citations per document. This analysis resulted in four clusters of 41 documents, represented in four colors ( Figure 4). The total number of citations across these 41 articles is 4422 (approximately 56%).

Cluster one, represented in red, has 13 articles named Sustainability and Wine, published between 2009 and 2016. The most cited article in this cluster was “Consumer attitudes regarding environmentally sustainable wine: an exploratory study of the New Zealand market place.” ( Forbes *et al.*, 2009), followed by “Millennial generation attitudes to sustainable wine: an exploratory study on Italian consumers” ( Pomarici and Vecchio, 2014). The other articles in this cluster discuss environmental impacts ( Amienyo *et al.*, 2015; Point *et al.*, 2012), the carbon intensity of wine distribution ( Cholette and Venkat, 2009), wine labeling and packaging ( Barber, 2010; Galati *et al.*, 2019) and environmental consciousness ( D’Amico *et al.*, 2016). Interestingly, a majority of studies have explored consumers’ attitudes toward sustainable/green/organic wines ( Forbes *et al.*, 2009; Mann *et al.*, 2012; Pomarici and Vecchio, 2014; Sogari *et al.*, 2015; Tait *et al.*, 2019; Wiedmann *et al.*, 2014).

Cluster two, represented in green has 11 “Wine Preferences and Choice” articles. The articles in this cluster were published between 2007 and 2012. The most cited article in this cluster is “A regional brand image and perceived wine quality: the consumer perspective ( Johnson and Bruwer, 2007). This cluster’s second most cited article compares international wine consumer choice ( Goodman, 2009). Few studies have investigated the reasons ( Charters and Pettigrew, 2008; Ritchie, 2007), perception ( Liu and Murphy, 2007), motivation ( Somogyi *et al.*, 2011), preferences ( Casini *et al.*, 2009), and determinants ( Camillo, 2012; Hussain *et al.*, 2007; Yu *et al.*, 2009) of wine consumption. Studies have also investigated the role of brand image ( Johnson and Bruwer, 2007) and country of origin ( McCutcheon *et al.*, 2009) in wine choice.

The third cluster, represented in blue, is named “Wine Consumer Behavior” and has nine articles. The articles in this cluster were published between 1997 and 2012. The most cited in this cluster is “The use of quality and reputation indicators by consumers: the case of Bordeaux wine” ( Landon and Smith, 1997). Studies in this cluster have investigated wine consumer behavior using different factors, such as country of origin ( Balestrini and Gamble, 2006; Bruwer and Buller, 2012; Dimara and Skuras, 2005; Orth *et al.*, 2005), information search ( Atkin *et al.*, 2007), wine involvement ( Barber *et al.*, 2007), grape vintage year ( Gil and Sánchez, 1997), and functional and production aspects ( Vrontis *et al.*, 2011).

The fourth cluster, represented in yellow is “Wine Consumer Insights: Tourism, Generations, and Preferences.” This cluster has eight articles on diverse aspects, such as wine tourists’ consumption behavior ( Bruwer *et al.*, 2013; Bruwer and Alant, 2009; Carlsen and Boksberger, 2015), sensory preferences ( Bruwer *et al.*, 2011), general differences ( Agnoli *et al.*, 2011; Mueller *et al.*, 2011), the role of demographics ( Bruwer *et al.*, 2012) and product involvement ( Bruwer and Huang, 2012). The most cited article in this cluster is “the hedonic nature of wine tourism consumption: an experiential view” ( Bruwer and Alant, 2009), followed by the article “consumer behavior and sensory preference differences: implications for wine product marketing” ( Bruwer *et al.*, 2011). The articles published in this cluster were published between 2009 and 2015.

### 3.4 Systematic review

**Figure 5.**
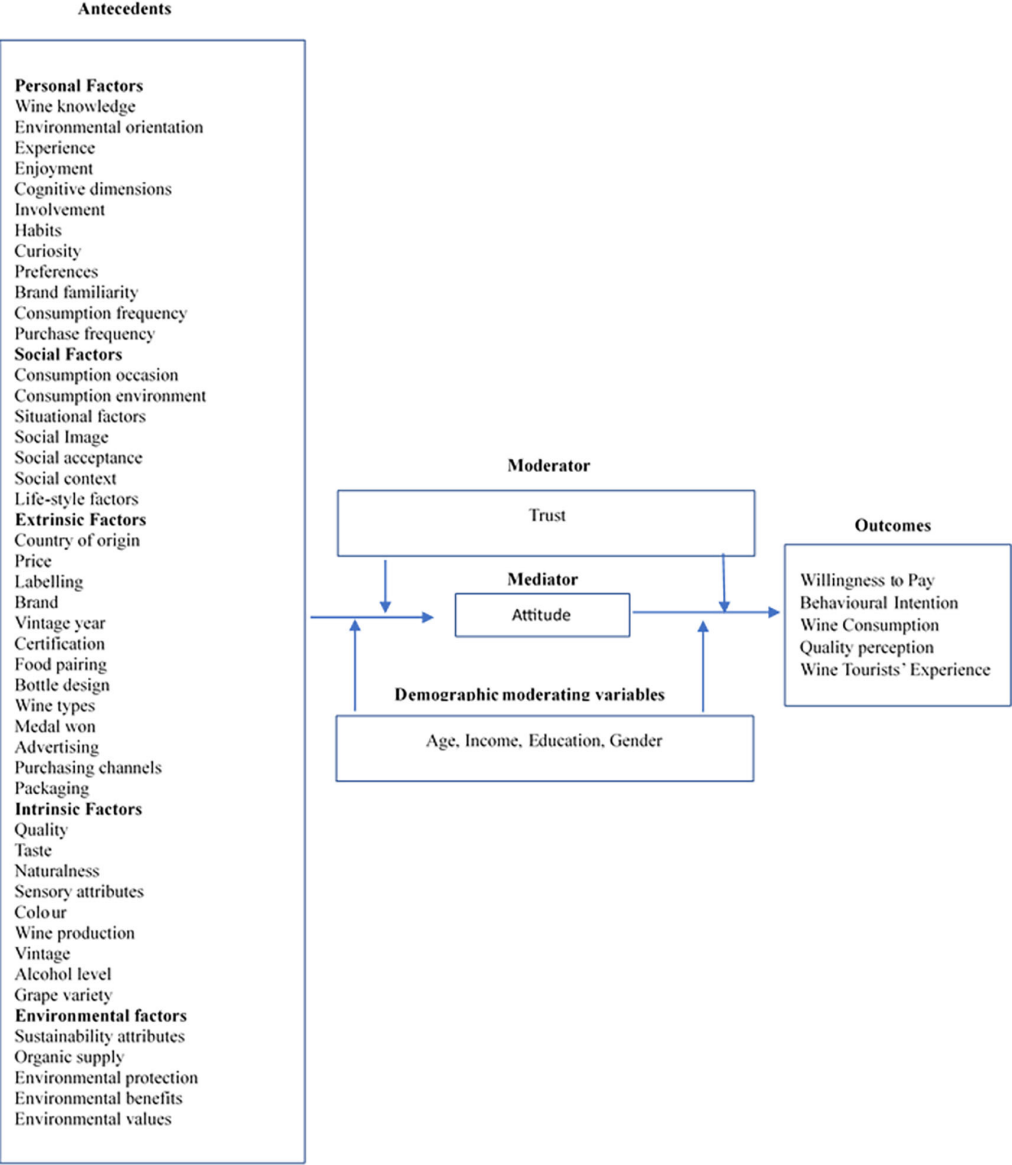
Antecedent outcome framework. Source: Author’s own.

The fourth objective of the study was to develop a conceptual framework based on the antecedents, mediators, and outcomes reported in the wine consumer literature. As mentioned in the methodology section, the documents forming bibliographical coupling were systematically reviewed to gain comprehensive insight into wine consumers’ literature. Three articles were excluded from this review because they did not pertain to wine consumer behavior. Figure 5 illustrates the framework developed by integrating the antecedents, mediators, and outcomes reported in the wine consumer literature.

3.4.1 Antecedents of wine consumer research

All the antecedents that predominantly influence wine consumer behavior are further categorized into five subcategories: Personal, Social, Extrinsic, Intrinsic, and Environmental.

Personal factors comprised variables related to the individual, such as wine knowledge, environmental orientation, experience, enjoyment, cognitive dimensions, involvement, habits, preferences, brand familiarity, consumption frequency, and purchase frequency. These factors can vary from person to person and can influence their preferences, choices, and decision-making processes when purchasing and consuming wine. For example, consumers with higher levels of objective wine knowledge are likelier to use intrinsic cues during wine purchase than extrinsic cues, such as country of origin ( Bruwer and Buller, 2012). Similarly, it is found that wine-tasting experience significantly correlated with attitude toward brand loyalty. Consumers having an enjoyable and memorable experience are more likely to repurchase and promote the wine brand to others ( Bruwer *et al.*, 2013).

Additionally, enjoyment, situational and lifestyle-related factors are found to be crucial ( Charters and Pettigrew, 2008). Specifically, enjoyment was critical across groups, irrespective of age and gender ( Charters and Pettigrew, 2008). Other personal factors that impact consumers’ consumption or purchase behaviors are consumption frequency, curiosity, purchase frequency, habits, and brand familiarity ( Balestrini and Gamble, 2006; D’Amico *et al.*, 2016; McCutcheon *et al.*, 2009).

A systematic review revealed that social factors, such as consumers’ living environment, situational factors, social image, social acceptance, and social context, were also found to be antecedents to wine consumption and purchase ( Agnoli *et al.*, 2011; Charters and Pettigrew, 2008; Iazzi *et al.*, 2019; Liu and Murphy, 2007; Orth *et al.*, 2005). For example, a study conducted among millennials revealed that consumers living in urban areas, being female and older, are likely to buy sustainable wine ( Iazzi *et al.*, 2019). The literature shows that wine consumption’s second most important factor is the situational factor ( Charters and Pettigrew, 2008). While social image impacted wine consumption ( Liu and Murphy, 2007), social context and acceptance were found to have an impact on wine preference ( Agnoli *et al.*, 2011; Orth *et al.*, 2005).

The third antecedent identified through the systematic review was extrinsic factors comprising variables such as country of origin, price, labeling, brand, vintage year, certification, food pairing, bottle design, wine types, medal won, advertising, purchasing channels, and packaging. Price is the most studied extrinsic variable by researchers ( Agnoli *et al.*, 2011; Atkin and Sutanonpaiboon, 2007; Balestrini and Gamble, 2006; Bonn *et al.*, 2016; Bruwer and Buller, 2012; Forbes *et al.*, 2009; Galati *et al.*, 2019; Gil and Sánchez, 1997; Johnson and Bruwer, 2007; Liu and Murphy, 2007; McCutcheon *et al.*, 2009; Orth *et al.*, 2005; Somogyi *et al.*, 2011; Tait *et al.*, 2019), followed by labeling ( Barber *et al*., 2007; Bruwer *et al*., 2011; Bruwer & Buller, 2012; Casini *et al*., 2009; D’Amico *et al*., 2016; Forbes *et al*., 2009, p. 200; McCutcheon *et al*., 2009; Sogari *et al*., 2015; Somogyi *et al*., 2011; Yu *et al*., 2009) and country of origin ( Atkin and Sutanonpaiboon, 2007; Bruwer and Buller, 2012; Gil and Sánchez, 1997; Johnson and Bruwer, 2007; Liu and Murphy, 2007; McCutcheon *et al.*, 2009, 2009). The other popular extrinsic variables studied were wine brands ( Casini *et al.*, 2009; D’Amico *et al.*, 2016; Johnson and Bruwer, 2007; Liu and Murphy, 2007; McCutcheon *et al.*, 2009; Yu *et al.*, 2009), bottle design, including packaging and wine stopper design ( Atkin and Sutanonpaiboon, 2007; Barber *et al.*, 2007; Bruwer and Buller, 2012; D’Amico *et al.*, 2016), wine types ( Atkin and Sutanonpaiboon, 2007; Bruwer and Li, 2007; Johnson and Bruwer, 2007; Somogyi *et al.*, 2011), and recommendation by peers and sales assistance ( Bruwer *et al.*, 2011; Bruwer and Buller, 2012; Casini *et al.*, 2009; McCutcheon *et al.*, 2009; Somogyi *et al.*, 2011; Yu *et al.*, 2009). Researchers have also explored the role of food pairing ( Casini *et al.*, 2009; Somogyi *et al.*, 2011; Yu *et al.*, 2009) and wine consumption in a few instances. The impact of medals won ( Casini *et al.*, 2009; Yu *et al.*, 2009), certification ( Sogari *et al.*, 2015), aesthetics ( Charters and Pettigrew, 2008), and purchasing channels ( Johnson and Bruwer, 2007) were also studied by researchers.

The fourth antecedent is identified as an intrinsic factor. It included characteristics that are inherent to the wine itself. Examples include quality ( McCutcheon *et al.*, 2009; Sogari *et al.*, 2015, p. 201; Tait *et al.*, 2019), taste ( Bruwer and Buller, 2012; D’Amico *et al.*, 2016), naturalness ( D’Amico *et al.*, 2016; Galati *et al.*, 2019), sensory attributes ( Bonn *et al.*, 2016; Galati *et al.*, 2019), color ( Bruwer and Buller, 2012; Mann *et al.*, 2012), vintage year ( Bruwer and Buller, 2012; Gil and Sánchez, 1997; McCutcheon *et al.*, 2009), alcohol level ( McCutcheon *et al.*, 2009; yu *et al.*, 2009), and grape variety ( Casini *et al.*, 2009; Goodman, 2009; McCutcheon *et al.*, 2009; yu *et al.*, 2009). Additionally, studies have also investigated the impact of environmental factors as the fifth antecedent comprising sustainability ( Tait *et al.*, 2019), organic supply ( Bonn *et al.*, 2016; D’Amico *et al.*, 2016; Galati *et al.*, 2019; Mann *et al.*, 2012; Wiedmann *et al.*, 2014), environmental protection, benefits, and values.

3.4.2 Mediators

The systematic review suggests that wine consumer researchers have rarely used any mediators. For example, the only study that used an exploratory approach to investigate wine consumers’ attitudes toward sustainable-labeled wine adopts an attitude that mediates the relationship between consumers’ beliefs about the wine and their wine consumption ( Sogari *et al.*, 2015). Furthermore, the findings of this study revealed that attitude toward sustainable-labeled wine is shaped by both environmental and quality beliefs about sustainable wine.

3.4.3 Moderators

Similar to mediators, wine consumer research studies have rarely adopted any moderators. Trust was the only moderator used in the study ( Bonn *et al.*, 2016). It is suggested that consumer perceptions of suppliers are the basis for deciding whether to believe a winemaker’s claim that their products and operations are sustainable ( Bonn *et al.*, 2016).

3.4.4.Demographic moderating variables

In addition to the moderating variable mentioned above, studies have examined the moderating role of demographic variables, such as age ( Sogari *et al.*, 2015), income, education ( Barber, 2010), and gender ( Atkin and Sutanonpaiboon, 2007; Barber, 2010).

3.4.5 Outcomes

The systematic review identifies several outcome variables discussed in the extant literature, namely, willingness to pay ( Barber, 2010; D’Amico *et al.*, 2016; Forbes *et al.*, 2009; Galati *et al.*, 2019; Pomarici and Vecchio, 2014; Skuras and Vakrou, 2002; Tait *et al.*, 2019), purchase intention, behavioral intention, wine selection ( Agnoli *et al.*, 2011; Bonn *et al.*, 2016; Bruwer *et al.*, 2011; Bruwer and Buller, 2012; Camillo, 2012; Casini *et al.*, 2009; Gil and Sánchez, 1997; Goodman, 2009; Liu *et al.*, 2017; McCutcheon *et al.*, 2009; Ritchie, 2007; Sogari *et al.*, 2015), wine consumption ( Balestrini and Gamble, 2006; Bruwer *et al.*, 2012; Charters and Pettigrew, 2008; Hussain *et al.*, 2007; Mann *et al.*, 2012; Mueller *et al.*, 2011; Somogyi *et al.*, 2011; Yu *et al.*, 2009), consumer wine quality perception ( Johnson and Bruwer, 2007), tasting experience ( Bruwer *et al.*, 2013), and wine tourists’ motivation ( Bruwer and Alant, 2009a).

3.4.6 Theoretical and methodological advancement

The fifth objective of the study was to explore the theoretical and methodological advancement in a wine consumer study. All 39 articles were subjected to systematic review, and the findings are discussed in the following section. Wine consumer scholars have used many theories, such as McFaden’s random utility theory ( Tait *et al.*, 2019), Lancasters’ characteristics theory ( Mann *et al.*, 2012; Tait *et al.*, 2019), and the theory of consumer behavior ( Camillo, 2012). Ego-involvement and Persuasion theory ( Bruwer and Huang, 2012), Age-Generational Cohort theory ( Bruwer *et al.*, 2011; Bruwer and Alant, 2009), VBA theory ( Sogari *et al.*, 2015), Brand loyalty ( Bruwer *et al.*, 2013), and Reputation model ( Landon and Smith, 1997). However, a majority of studies remain atheoretical.

The systematic review suggests that the majority of the researchers contributed to the wine consumer literature through quantitative studies (N=30), followed by mixed methods (N=5) and qualitative methods (N=4). Only one case study was found.

### 3.5 Future research directions

**Figure 6.**
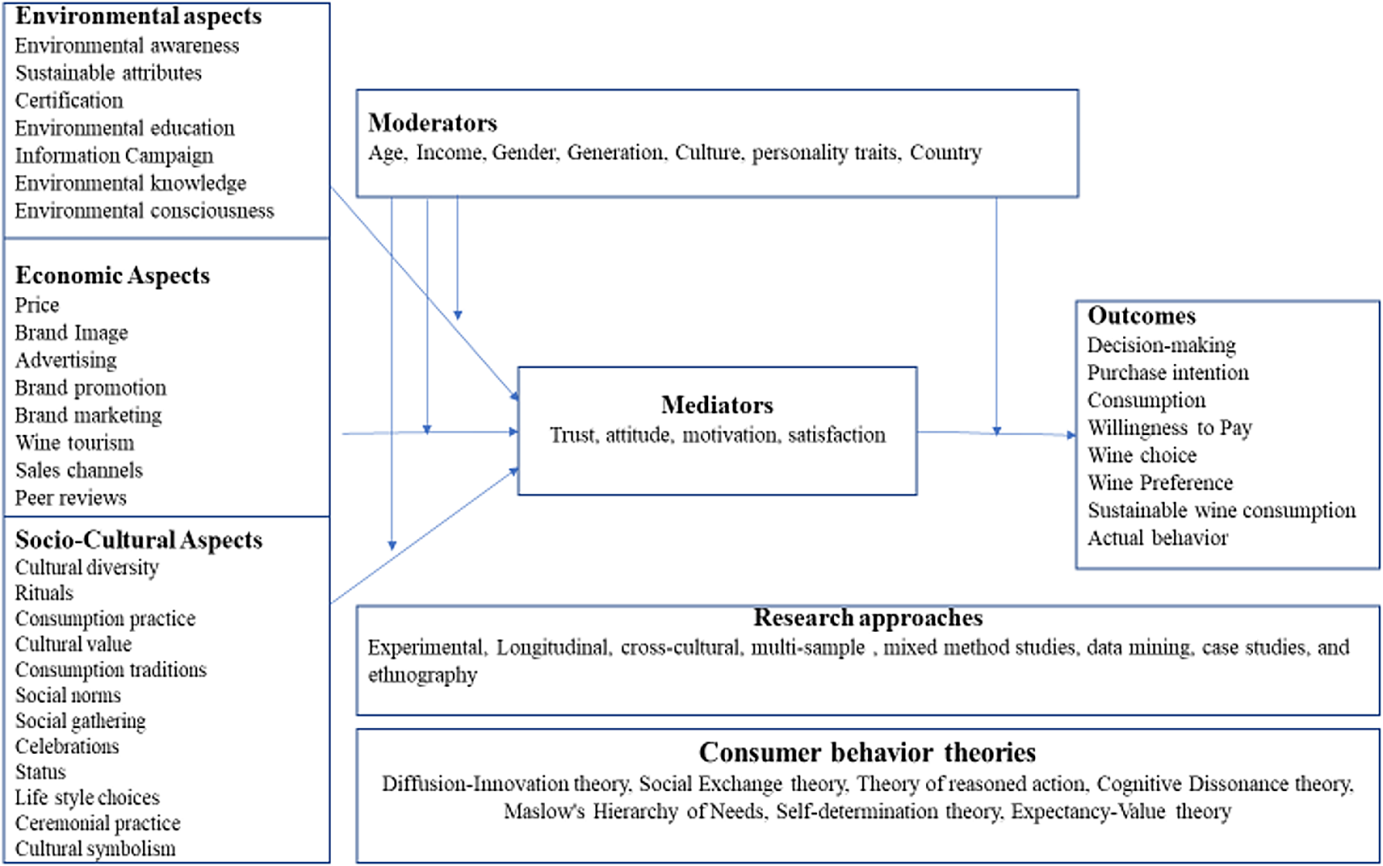
Proposed theoretical framework. Source: Author’s own.

This study’s sixth and final objective is to recommend future research directions in wine consumer research. Although the previous wine literature sheds light on some of the critical areas related to consumers, it is found that there is a scope for future research related to environmental, economic, and sociocultural issues. Some crucial areas within these issues are identified ( Figure 6), and future directions are discussed in the following section.

3.5.1 Antecedents

Environmental aspects

By addressing environmental issues related to wine consumption, producers can contribute to the industry’s overall sustainability, minimize ecological impacts, and meet the growing demand for environmentally friendly products. Additionally, taking proactive measures to address these issues helps ensure wine-producing regions’ long-term viability and resilience in the face of climate change and environmental challenges. Therefore, future studies should focus on some environmental aspects, such as wine consumers’ environmental knowledge, education, consciousness, and impact on their purchase intention, preference, and willingness to pay for sustainable wine. Researchers can also focus on the role of sustainability attributes on wine consumption preferences, choices, and consumption. Another potential area of research is the role of certification and its impact on wine-purchasing behavior. Future studies can also measure the role of environmental attributes on actual wine consumption.

Economic aspects

The economic attributes of wine consumer behavior refer to the factors and considerations influencing consumers’ choices and behaviors about purchasing and consuming wine. Future researchers can consider key economic attributes: price, brand image, brand communication, advertising, brand promotion, and sales channels. For example, a recent study conducted to understand the impact of brand communication and brand image on brand preference revealed that both variables positively affected brand preferences ( Gómez-Rico *et al.*, 2023). Wine sales channels are another critical variable to consider in future studies. There is a correlation between the usage of sales channels and per capita consumption and wine preferences ( Szolnoki and Hoffmann, 2014).

Sociocultural aspects

Sociocultural aspects play a significant role in wine consumption. For example, wine is often deeply rooted in cultural traditions and heritage ( Black and Ulin, 2013). It is associated with specific regions, countries, and communities, reflecting their history, customs, and social identity ( Trivellato *et al.*, 2014). Wine consumption is also linked to social and emotional well-being ( Bauer and Mills, 2021). It is also associated with special occasions, rituals, and ceremonies, adding to its sociocultural significance. Therefore, future research can focus on the sociocultural aspects of wine, such as cultural diversity, rituals, consumption practice, cultural value, consumption traditions, social norms, social gathering, celebrations, status, lifestyle choices, ceremonial practice and cultural symbolism, and its impact on consumer preference and choices. Future research can also focus on the impact of sociocultural aspects on the emotional and social well-being of wine consumers.

3.5.2 Mediators

Future studies can also focus on mediating variables such as attitude, motivation, trust, and satisfaction. For example, attitudes can mediate the relationship between wine-related stimuli (e.g., marketing messages, product attributes) and consumer behavior (e.g., purchase intentions, consumption, willingness to pay, wine preferences, and brand loyalty). Similarly, wine consumers’ motivation can also be important in determining wine consumption behavior. It can influence wine consumption occasions, preferences for certain types of lifestyles, and engagement in wine activities, such as wine tourism. Trust is another important factor in consumer behavior. behavior Trust in wine brands, producers, or retailers can mediate the relationship between wine-related stimuli and consumers. Trustworthy wine-related information and certifications can enhance purchase intentions. Future studies can also examine the mediating role of consumer satisfaction. Consumer satisfaction can mediate the relationship between brand image and brand loyalty. Satisfied consumers are likelier to exhibit repeat purchases, positive word-of-mouth, and brand loyalty.

3.5.3 Moderators

Moderator variables play a crucial role in understanding the nuances of wine consumption and how it varies across different individuals and contexts. Some moderators that can be considered in future studies are age, income, gender, generation, culture, personality traits, and country.

Age can moderate wine consumption patterns, preferences, and behaviors. For example, younger consumers may be more experimental and open to trying different wine styles, while older consumers might have more established choices and seek specific wine characteristics. Furthermore, age can also influence the context in which wine is consumed, such as social occasions or personal relaxation. Another significant moderator is income. Higher-income individuals may be more likely to purchase premium/luxury/sustainable wines. Income can also impact the frequency and occasions of wine consumption. Gender is another critical moderator that can be considered in future studies. Research suggests that gender differences exist in wine choice, with variations in the preference for specific wine styles, labeling aesthetics, or marketing messages ( Alpeza *et al.*, 2023).

Additionally, gender can influence the social context of wine consumption and the roles individuals play in wine-related activities. Future research can also focus on the generational differences in values, wine knowledge, consumption preferences, habits, and frequency by their respective social and historical contexts. The moderating role of personality traits can also be investigated in future studies. For example, individuals with higher innovativeness may be more inclined to try different wine varieties. The cultural context of the country can moderate wine consumption practices. For example, a comparative study between new and old wine countries can be considered. Each country has its unique wine culture and consumption norms. Factors such as wine availability, legal regulations, and historical wine traditions can shape consumer behavior within a country.

3.5.4 Outcomes

Despite the extensive focus of researchers on various outcomes, including willingness to pay, behavioral intention, wine consumption, quality perception, and tourists’ experience, this research suggests that there remains ample opportunity for further investigation. Examples include wine choice, preferences, sustainable wine consumption, brand loyalty, and emotional/social well-being. Furthermore, future studies can also focus on actual wine consumption. For example, research on well-being can include the role of wine consumption across different social occasions/gatherings. Understanding wine consumption’s emotional and social dimensions can provide insights into its broader impact on individuals’ well-being.

3.5.5 Theoretical directions

It is recommended that future studies use the consumer decision-making process (CDMP) model, which provides an understanding of wine consumers’ various decision-making stages, such as problem recognition, information search, evaluation of alternatives, purchase, and postpurchase evaluation. These models can also be applied to investigate consumers’ choice of wine products, considering price, brand, taste, labeling, and recommendations. In addition, this research explores several theories that can be adopted in the future and are discussed below.

Experiential consumption theory ( Holbrook and Hirschman, 1982) emphasizes the significance of experiential aspects, such as sensory pleasure, emotions, and fantasies, in consumer behavior and can also be considered by wine consumer scholars. This theory argues that experiential or hedonic dimensions shape consumer preferences and satisfaction. Since its inception, experiential consumption theory has been widely adopted and expanded upon by scholars in consumer behavior. However, this theory is sparingly adopted in wine consumer studies ( Bruwer and Alant, 2009).

Another theory that can be considered in wine consumer studies is the diffusion of innovation theory ( Rogers, 2010). This theory describes how new products, ideas, and behaviors gradually spread among the population. It contends that adoption starts with innovators and early adopters and then spreads through the people to the early and late majority. In wine consumer studies, this theory can be adopted to examine the adoption of new wine products, profiling wine consumers based on the adoption of new wine products and the acceptance of new wine products, practices, or technologies among consumers. This theory can also be adopted to investigate the role of opinion leaders in adopting new wine products.

This review identifies the theory of planned behavior (TPB) as a valuable framework for assessing the behavioral antecedents of wine consumption. It is found that TPB better predicts wine consumption in restaurants than Theory of Reasoned Action (TRA) ( Agnoli *et al.*, 2016; Agnoli and Outreville, 2023). The TPB ( Ajzen, 1987) is a psychological theory that explains and predicts human behavior in various social contexts. This theory suggests that attitudes, subjective norms, and perceived behavioral control influence people’s actions.

Cognitive dissonance theory ( Festinger, 1957) is a social psychology theory that seeks to explain the psychological discomfort (cognitive dissonance) that arises when individuals hold conflicting beliefs, attitudes, or values. This theory has been used in the consumer behavior literature to measure the cognitive dissonance of medical tourists ( Majeed *et al.*, 2022) and travel behavior ( Kah and Lee, 2016). In a wine consumer study, this theory is used to understand how tensions may arise for individuals approaching each experience and where to avert perceived risks ( d’Ament *et al.*, 2022). However, this theory can be used in other areas of wine consumption, such as dissonance between price and quality, brand loyalty and consumer preference, wine recommendations and consumption experience.

According to Maslow’s hierarchy of needs ( Maslow, 1943), individuals are motivated to fulfill their needs sequentially, from the most basic to higher-order psychological needs. This theory was used to segment wine consumers ( Divine and Lepisto, 2005) and understand wine tourists’ motivation ( Park *et al.*, 2008). Future studies can use this theory to understand the relationship between psychological needs, safety, emotional experience, social status, and wine consumption behavior.

Self-determination theory (SDT) ( Deci and Ryan, 1985) is one of the psychological theories of motivation that can be considered in future wine consumption studies. The theory focuses on the innate psychological needs that drive human behavior and the conditions that foster personal growth and well-being. This theory is used to understand the relationship between motivation and wine club attributes ( White and Thompson, 2009) and attitude toward domestic wine ( Ye *et al.*, 2017). However, future wine consumer studies can further explore this theory to understand the role of intrinsic/extrinsic factors on consumer behavior. For example, how can social interactions, such as wine festivals or wine-tasting events, enhance wine consumers’ experience? How can wine education contribute to wine consumers’ competence and overall experience?

Expectancy-value theory (EVT) ( Atkinson, 1957) is another motivational theory that wine researchers can use to understand how expectancies and values about different antecedents, such as environmental, economic, and sociocultural attributes. For example, how consumers’ expectations and values regarding the health benefits of wine shape their purchase and consumption frequency. Wine consumer scholars have adopted this theory to explore the relationship between experience and current consumption behavior ( Melo *et al.*, 2010, p. 202) and to predict wine consumption ( Cox, 2009).

3.5.6 Methodological directions

Longitudinal studies play a crucial role in enhancing the wine consumer literature by providing valuable insights into consumer behavior and its evolution. It helps in pursuing consumers’ preferences and consumption trends and changes in the decision-making process. Therefore, future studies can adopt a longitudinal study approach to investigate how consumers’ preferences for different types of wines evolve. Studies can also explore the long-term impact of wine education on consumers’ knowledge and competence in wine selection. Other research approaches recommended are experimental (for example, wine label information and purchase intention), cross-cultural (between new world wine and old wine countries), mixed method, case studies, such as the impact of wine tourism on local wineries’ business, and ethnographical studies.

## 4. Implications

This integrative review yields several noteworthy implications for future research and practical applications. First, the publication trends reveal the growth trajectory of wine consumer literature from 1985 to 2023. Second, the performance analyses reveal the most influential authors regarding the number of publications, citations, active years, and H-index. Third, this study also identifies the most influential works in the wine consumer literature. This finding serves as a reading list for future researchers to understand the studies’ theories, methods, research context, findings, and implications. Fourth, this study contributes to the literature by identifying the most influential journals and productive countries. Fifth, the science mapping techniques (keyword co-occurrence and bibliographic coupling analysis) used in this study provide a holistic knowledge map of wine consumer literature. This visualization helps future researchers identify the topics and areas of wine consumer literature that have already received much attention.

Similarly, the systematic review also has several implications. First, it summarizes the literature on wine consumers regarding antecedents, mediators, moderators, and outcomes integrated in the extant literature. Based on the systematic review, this study classifies antecedents of previous studies into five subcategories and provides an integrative framework. Second, it also summarizes the extant literature in terms of theoretical and methodological advancement. Second, this study also highlights the theoretical and methodological advancements in the wine consumer literature. This helps future researchers identify the knowledge gaps in terms of theory and research methods adopted by wine consumer scholars. Third, based on the identification of antecedents, mediators, moderators, and outcomes, this study proposes a framework for future directions. The framework also provides theoretical and methodological recommendations for future study.

## 5. Conclusions and limitations

Integrating bibliometric analysis and systematic review in the wine consumer literature has provided insights into the present state of research and highlighted critical trends, knowledge gaps, and significant clusters. The bibliometric analysis demonstrated the expansion of research on wine consumer behavior, demonstrating a growing scholarly interest in the preferences, behavioral intention, willingness to pay, wine quality perception, and decision-making processes of wine consumers. It highlighted the areas of emphasis and the intellectual network within the subject by identifying important authors, influential publications, the most contributing countries, and the most impactful journals. It also identifies critical areas of wine consumer research. Thus, these analyses not only give a picture of the current state of the literature but also lay the groundwork for future research endeavors.

In contrast, the systematic review provided a comprehensive analysis of the content and findings of the selected papers. It identified the antecedents, mediators, moderators, and outcomes of the wine consumer literature by synthesizing current research. In addition, the article discusses the methodology and theoretical frameworks utilized in the wine consumer literature. This combination helped to uncover knowledge gaps and inconsistencies, paving the way for future research endeavors. Furthermore, it provided insights into the personal, social, extrinsic, intrinsic, and environmental factors that influence wine consumption behavior. Combining quantitative analysis of publication trends and citation metrics with qualitative synthesis of study findings, this multidisciplinary strategy has comprehensively examined the current literature. By expanding upon the results of this study, researchers can continue to contribute to the growth of wine consumer behavior knowledge, which will ultimately help the wine business and wine consumers.

This study is based on referred English articles from the Scopus data collection. Therefore, the findings may not reflect papers in other databases, such as the Web of Science of Google Scholars. Thus, future research may consider data from multiple sources to provide comprehensive insight into the wine consumer literature. Since the inclusion of articles in the systematic review is based on the bibliographic coupling analysis, it is challenging to claim absolute inclusion of all articles related to wine consumption.

## Data Availability

Figshare: Wine consumer behavior New, https://doi.org/10.6084/m9.figshare.25157096.v3 ( Mallya *et al*., 2024a). This project contains the following underlying data:
• scopus (3).csv Figshare: PRISMA checklist and flowchart for: ‘Wine Consumer Studies: Current Status and Future Agendas’. https://doi.org/10.6084/m9.figshare.25200755.v1 ( Mallya *et al*., 2024b) Data are available under the terms of the Creative Commons Zero “No rights reserved” data waiver (CC0 1.0 Public domain dedication). The VOSviewer software is available freely.
