# Supplementary material for: Wine Consumer Studies: Current Status and Future Agendas
Source: F1000Res. 2024 Mar 27;13:228. [Version 1] doi: 10.12688/f1000research.146631.1 (PMC11727852; doi:10.12688/f1000research.146631.1)
Supplement: Peer review history

# Reviewer report: Reviewer response for version 1

**DOI:** 10.5256/f1000research.160735.r354257

**Published:** 2025-01-11

**Authors:** Jighyasu Gaur

**Affiliations:** 1 Indian Institute of Management Raipur, Raipur, Chhattisgarh, India

The research conducted by the authors is timely and relevant. The authors have done a decent job and identified critical areas. 

 Please find below my detailed review report.

 - The research conducted by the authors is both timely and relevant, addressing an important topic that holds significance in the current academic or professional landscape. 

 - The authors have identified critical areas that contribute meaningfully to the ongoing discourse in their field of study. 

 - Their efforts demonstrate a good understanding of the subject matter and a commendable ability to pinpoint issues that require attention or further exploration.

 - However, I would like to draw attention to a few grammatical errors present in the manuscript, which should be corrected to enhance the overall readability and professionalism of the work. 

 - These minor issues, once addressed, will ensure that the research is presented in the best possible manner. I recommend the authors carefully proofread the document or use professional editing tools or services to rectify these errors.

 Overall, the paper is well-structured and presents valuable insights. I wish the authors all the best in revising their work and look forward to seeing the final version published.

Are the rationale for, and objectives of, the Systematic Review clearly stated?

Yes

Is the statistical analysis and its interpretation appropriate?

Yes

If this is a Living Systematic Review, is the ‘living’ method appropriate and is the search schedule clearly defined and justified? (‘Living Systematic Review’ or a variation of this term should be included in the title.)

Yes

Are sufficient details of the methods and analysis provided to allow replication by others?

Yes

Are the conclusions drawn adequately supported by the results presented in the review?

Yes

Reviewer Expertise:

Supply chain, consumer research

I confirm that I have read this submission and believe that I have an appropriate level of expertise to confirm that it is of an acceptable scientific standard.

# Reviewer report: Reviewer response for version 1

**DOI:** 10.5256/f1000research.160735.r354256

**Published:** 2025-01-09

**Authors:** Amol Dhaigude

**Affiliations:** 1 Department of Operations Supply Chain Management, S P Jain Institute of Management and Research, Mumbai, Maharashtra, India

The manuscript presents an integrative review of the literature on wine consumers' behavior, employing bibliometric analysis and systematic review methodologies. The manuscript is structured into sections covering the introduction, methods, results and discussion, and conclusions, which aim to map the existing body of research, identify gaps, and propose future research directions. While the effort to provide a comprehensive overview is evident, there are several areas where improvements could significantly enhance clarity, coherence, logical progression, flow, and organization.

 First, the flow of information.

 The transition from introducing wine as a cultural icon to the specifics of consumer behavior research feels abrupt. There is a leap from general admiration of wine to detailed methodologies without a smooth transition that guides the reader through the rationale behind the chosen methods. For example, "Since ancient times, wine has been admired as a cultural icon... Therefore, this integrative review aims to provide the current state of knowledge of wine consumers' behavior."

 Second, Coherence between paragraphs. The coherence between sections is weakened by the jump from discussing the motivation behind the review to detailed methodological descriptions. A brief overview of the study's structure could enhance coherence. Examples: "Such omissions motivated the researchers... ….. Methods."

 Third, the logical progression: The logical progression from identifying research gaps to proposing a methodological approach is somewhat obscured by dense paragraphs and a lack of clear signposting. Examples:  "Such omissions motivated the researchers... an integrating these approaches offers a unique opportunity..."

 Fourth, regarding the clarity of information, some sections are overloaded with details that could be streamlined for clarity, especially the methods and results sections.

 Fifth, some inconsistencies are found. For example, different forms of this word have been used in the text. For example, 'co-occurrence' and 'cooccurrence', 're-visit' and 'revisit'. Please pick one style and use it consistently throughout the text.

 The above feedback could significantly improve its readability and impact.

 All the best.

Are the rationale for, and objectives of, the Systematic Review clearly stated?

Yes

Is the statistical analysis and its interpretation appropriate?

Yes

If this is a Living Systematic Review, is the ‘living’ method appropriate and is the search schedule clearly defined and justified? (‘Living Systematic Review’ or a variation of this term should be included in the title.)

Partly

Are sufficient details of the methods and analysis provided to allow replication by others?

Yes

Are the conclusions drawn adequately supported by the results presented in the review?

Yes

Reviewer Expertise:

Bibliometric analysis, SLRs, Case Method, SCM, PLS-SEM

I confirm that I have read this submission and believe that I have an appropriate level of expertise to confirm that it is of an acceptable scientific standard.
